# Toward cross-cultural emotion detection: the Indian spontaneous micro-expression dataset

**DOI:** 10.3389/fpsyg.2025.1656104

**Published:** 2025-10-16

**Authors:** Riya Mishra, Braj Bhushan, K. S. Venkatesh

**Affiliations:** ^1^Department of Humanities and Social Sciences, Indian Institute of Technology Kanpur, Kanpur, India; ^2^Department of Electrical Engineering, Indian Institute of Technology Kanpur, Kanpur, India

**Keywords:** micro-expression, facial action coding system, emotion recognition, behavioral database, cross-cultural emotion

## Introduction

Micro-expressions (ME) are brief, involuntary, and subtle facial expressions. These expressions typically last between 40 and 500 ms (Ekman, 2006). These fleeting expressions are crucial in revealing genuine emotional states, often surfacing when an individual attempts to mask their true feelings, consciously or unconsciously (Ekman and Friesen, 1969). Unlike Macro-facial expressions, which can be controlled or manipulated, MEs are difficult to fake, indicating a genuine emotional state (Matsumoto and Hwang, 2011). The study of MEs has significant implications across multiple domains, including clinical psychology, security, and human-computer interaction, as they offer insights into individuals' deeper, often hidden psychological states (Ekman, 2003). Understanding and interpreting these expressions can thus enhance communication and empathy, improving interactions in both social and professional settings.

Various databases have been developed to facilitate the study of MEs, providing researchers and technologists with essential data for training individuals or algorithms and conducting analyses. For instance, the USF-HD (Shreve et al., 2011) and Polikovsky databases (Polikovsky et al., 2009) focus on posed expressions. Similarly, the York DDT (Warren et al., 2009) and SDD (Pfister et al., 2011) used Lie-generation method, primarily featuring Western populations. ME databases like CASME (Yan et al., 2014a), CASME II (Yan et al., 2014b), and CAS (ME)^2^ (Qu et al., 2017) databases capture spontaneous expression with high-resolution recordings of spontaneous MEs from East Asian participants, focusing on the fine details of these brief emotional cues (Yan et al., 2014a,b). Each of these databases contributes valuable insights; however, they also come with notable limitations that influence the scope and generalizability of research in the field.

### Challenges in existing ME databases

While these databases have advanced the study of MEs, they exhibit significant limitations, particularly in terms of cultural diversity and representation. Most existing datasets predominantly focus on Western or East Asian populations, leaving other cultural groups, such as Indian populations, underrepresented. This lack of diversity raises concern about the generalizability and cross-cultural validity of the research findings. Research has shown that cultural context heavily influences how facial expressions are displayed and perceived (Matsumoto, 1993; Matsumoto et al., 2012).

Cultural context is fundamental in shaping the display and interpretation of emotions, and this variability can lead to misunderstandings in cross-cultural interactions. Western cultures, for instance, tend to emphasize the expression of emotions and may interpret direct facial cues as straightforward reflections of internal states (Matsumoto, 2006). In contrast, Asian cultures often prioritize social harmony, and facial expressions may convey less overt emotional information, relying instead on subtle cues integrated with broader contextual understanding (Jack et al., 2009). Such cultural differences are profound, as they highlight that emotional expressions are not universal but rather influenced by cultural norms and practices. This variability poses challenges for developing emotion recognition systems that can function accurately across diverse populations.

Existing databases, which predominantly feature Western or East Asian participants, may not be sufficient for developing systems that cater to other cultures. A brief summary of each dataset is also provided to contextualize their methodology, induction paradigms, and cultural focus, with full references included (Table 1).

**Table 1:** Comparative summary of existing micro-expression (ME) databases, including participant ethnicity, sample size, annotation method, emotion induction paradigm, emotional labels used, and feature selection approach.

**ME database**	**Participants (ethnicity)**	**Sample**	**Annotation**	**Emotion induction method**	**Emotional label**	**Feature selection method**
Canal-9 (Vinciarelli et al., 2009)	N/A	24 sequence	–	Political TV debates	No emotional label	Role-I based (in debates)
YorkDDT (Warren et al., 2009)	9 (Caucasians)	18	FACS	Lie generation	6 basic emotion	6 basic emotion
SDD (Radlak et al., 2015)	101 (N/A)	183	–	Lie generation	Deceiver & truth teller	Non-verbal potential cue (facial & eye movement)
MMEW (Ben et al., 2021)	36 (N/A)	300 (234 from 6 basic E)	FACS	N/A	6 basic emotion	6 basic emotion + others
USF-HD (Shreve et al., 2011)	N/A	100	ME/non-ME	Acting	6 basic emotion	6 basic emotion
Polikovsky (Polikovsky et al., 2009)	10 (Caucasians, Asian, Indian)	42	FACS	Acting	6 basic emotion + contempt	6 basic emotion + contempt
CASME I (Yan et al., 2014a)	19 (Chinese)	195	FACS	Neutralization	6 basic emotion	6 basic emotion
CASME II (Yan et al., 2014b)	35 (Chinese)	247	FACS	Neutralization	6 basic emotion	6 basic emotion
CAS (ME)^2^ (Qu et al., 2017)	22 (Chinese)	57 (ME)	FACS	Neutralization	6 basic emotion + 4 (P, S, N, O)	6 basic emotion + (P, S, N, O)
SMIC (Li et al., 2013)	20 (Caucasians, Asian, African)	164	Self-reported	Neutralization	P, N, S & H, SU, D, F, Sa	P, S, N & H, Su, D, F, Sa
SAMM (Davison et al., 2016)	32 (13 ethnic group)	159 (ME)	FACS	Neutralization	6 basic emotion	6 basic emotion
ISMED	120 (Indian)		FACS & self-reported	Neutralization	6 basic emotion- fear	6 basic emotion + neutral emotion

The table highlights the cultural diversity and methodological variability across datasets, underscoring the relevance of the Indian Spontaneous ME Database (ISMED).

Algorithms trained on these datasets may struggle when applied in different cultural contexts, leading to misinterpretations and inaccuracies. For instance, an expression considered a sign of discomfort in one culture might be perceived as an expression of agreement in another. Such discrepancies demonstrate the necessity of culturally diverse databases to enhance the reliability and precision of ME detection, particularly as these technologies are increasingly deployed in multinational, multicultural contexts like global security checkpoints, customer service environments, and cross-cultural therapeutic settings.

The current database (Indian Spontaneous Micro-expression Database: ISMED) tries to diversify the available data by providing another culturally diverse resource for ME research. With its vast population and rich cultural diversity, India offers a unique opportunity to study how emotions are expressed and perceived within its context. The objective of this study is to fill this gap by documenting spontaneous MEs from Indian participants, offering a comprehensive dataset that captures the nuanced facial expressions across six basic emotions—ranging from happy, disgust, anger, sadness, and fear to surprise.

By addressing existing databases' current limitations and highlighting the significance of cultural factors in emotion research, ISMED represents a critical step forward in non-verbal behavior studies. By emphasizing the need for culturally sensitive and representative research, ISMED contributes to a more inclusive understanding of human emotions and supports creating more accurate, effective, and culturally responsive emotion recognition systems.

## Methodology

### Participants

One hundred and twenty-seven participants (64 male and 63 female) of Indian ethnicity aged 20- 35 years volunteered to participate in this study. The mean age of the participants was 26.57 years (SD 4.32). The mean age of the male and female participants was 26.17 years (SD = 4.17) and 26.97 years (SD = 4.45), respectively. Participants with facial occlusions, such as mustaches, beards, spectacles, or makeup, were excluded to ensure clear facial visibility. The inclusion criteria emphasized individuals with low cheek fat and minimal wrinkles, leading to the selection of young adults aged 20–35. To maintain gender balance, the sample included 64 males and 63 females. Participants were carefully selected to represent a diverse range of socio-economic statuses (from lower to upper class) and cultural contexts from various regions of India. The study protocol was reviewed and approved by the Institute Ethics Committee (IITK/IEC/2021-22/II/26).

### Experimental set-up

#### Camera & light

The video was recorded by the GoPro7 camera with a 4k sensor. It records the participant's video at 240 frames per second with 1080 pixel resolution while using the HEVC video compression option. In order to maximize storage, this mode shrinks the file size.

Lighting is an issue for high-speed camera recording, as many light sources in lab use alternating current that refreshes regularly and create a flicker in the recording. Two rectangular LED screen bar lights were positioned on either side of the monitor to avoid it. It ensured consistent lighting on the face and minimized shadow.

### Emotion inducement stimuli

The Audio-visual video clips were used as stimuli to induce emotion in this study. These video clips were taken from the internet. Most of these video clips were taken from Indian movies, web series, plays, and YouTube shorts. Initially, 210 video clips were chosen by the experimenter for the emotion-labeling and rating study. These 210 video clips contain 30 video clips of each emotion (Anger, Disgust, Fear, Happy, Neutral, Sad, Surprise).

Eleven participants (Age range = 24–32 years, Mean age = 30, SD = 3.04), among them five were male (Mean = 29.4, SD = 2.24) and six were female (Mean = 26.83, SD = 1.94), These participant were not otherwise involved in the entire study, were involved in the emotion-labeling and rating study, to ensure inter-rater reliability.

In the labeling-rating task 210 video clips were first converted into “.avi” format and then were randomly presented using the Psychopy v2.0 on a 23-inch HPW2371d LED monitor screen. The Neutral-Emotion-Neutral (N-E-N) paradigm was used. In each trial first a fixation cross (1 s) of black color on white background then audio-visual video clips and lastly two questions; one of labeling (What emotion do you feel?) with seven forced choice option (Anger, Disgust, Fear, Happy, Neutral, Sad, Surprise) and rating (What was the intensity of felt emotion?) on a five-point were shown. All 210 video-clips were presented in three sessions on three different days. In this experiment a 20-inch monitor was used in a dimly lit room.

After the labeling and rating task, from 210 video clips, 52 video clips were selected (Angry-7, Disgust-8, Fear-6, Happy-8, Neutral-7, Sad-8, Surprise-8). These video-clips have a labeling consistency of 90% and an average rating of 4 or above. The selected 52 video clips will be further used in the study as emotion-inducing stimuli.

It is important to note that the stimulus category was used only to ensure balanced representation of all emotions and to avoid experimental bias. The final labeling of micro-expression instances did not rely on stimulus identity but was determined through subsequent AU-based and FACS-based validation (see below).

### Equipment & experimental set-up

The experiment was set-up in a soundproof room with audio-visual clips running on speaker mode. A separate room was used for the experiment running purpose to avoid any unnecessary surrounding disturbance. The experiment was set-up on a 58 × 58 cm monitor, and the resolution was set at 1920 × 1080 pixels. A GoPro camera was mounted in the middle of the upper edge of the monitor. For the lightning purpose (1.2 × 2.8 cm), an LED light was used in on both sides of the monitor panel to cancel out shadows.

**Figure 1:**
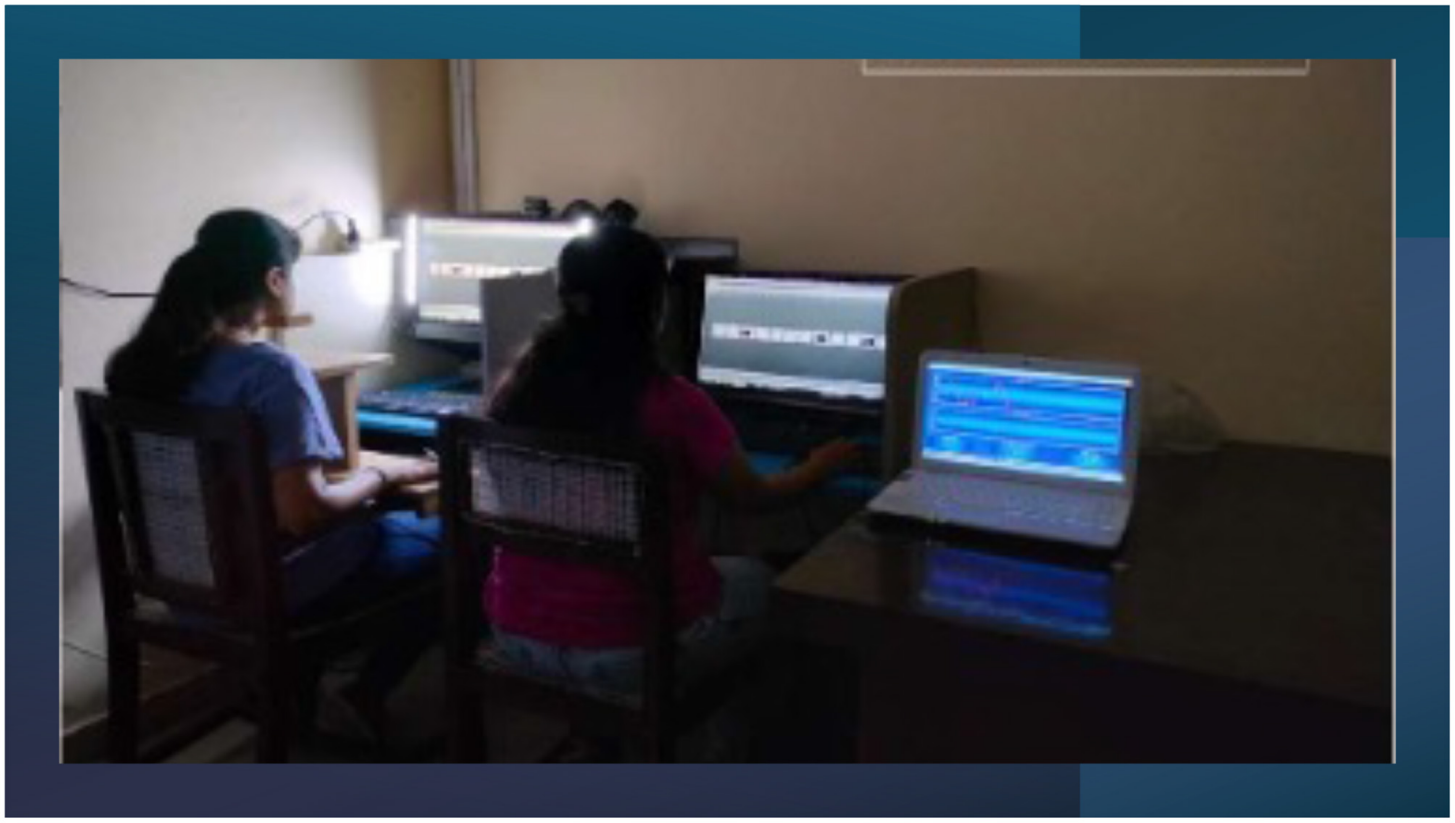
An experimental set-up shows the participant seated in front of the stimulus display monitor with lighting and a GoPro camera positioned for optimal ME recording. The experimenter monitors the set-up from an adjacent station to ensure minimal intrusion and consistent environmental control.

All extra lights (like- room lights or lights from other sources) were switched off during the experiment. An experimental set-up is shown in Figure 1.

The N-E-N paradigm was used to design the experiment. In each trial, a fixation cross (“+” sign) for 1 s, audio-visual video clips of 2 s to 1.5 min, and two questions of labeling and rating the video clips (each of 5 s) were shown. All the 52 video clips were shown in two sessions, each containing 26 trials. A 30 min time gap was given between the two sessions.

### Emotion inducement experiment procedure

Participants were first introduced to the experiment-room setting to establish rapport. They were briefed about the experiment, which involved watching video clips while maintaining a neutral facial expression to ensure the camera did not record any emotional expressions. Each participant was informed that the winner of the expression suppression game would receive 50 rupees. However, at the end of the experiment, all participants received 50 rupees as compensation for their time. They were also briefed on their rights, including the option to leave the experiment at any point if they felt uncomfortable. Any questions from participants were addresse before proceeding.

Each participant signed a hard copy of the consent form before the experimental session began. Once participants consented and wanted to participate in the study, we checked their eyesight, adjusted the audio level to a comfortable setting, and ensured the chin rest was positioned according to their heights. The side panel light brightness was kept constant to avoid variations in video quality.

Next, we attached interoceptive sensors to record their heart rate, respiration rate, and galvanic skin response. Participants were then asked to sit comfortably, while the experimenter made sure the distance was 65 cm between their eyes and the screen. The distance of 65 cm was maintained to ensure a constant visible active area. We proceeded with a demo session with the chin rest and eye-tracker sensors calibrated, and the high-speed recording camera adjusted to capture the desired frame.

The demo session included an instruction page and two trials; each consisted of a fixation cross, a video clip, and labeling-rating questions. Following the demo, we checked with participants to ensure they were comfortable continuing with the experiment. Upon their agreement, we commenced the first main session, lasting 15 min. After a 30-min break, the second session, lasting 15 min, began. During both main sessions, the experimenter was out of sight to maintain privacy and minimize experimenter presence bias.

### Video-acquisition result

Each participant gave 30 min of data. We started with 127 participants' data, 7 participants data were not included due to some technical glitches and lighting issues. One hundred and twenty participants' data were acquired and used for further processing.

### ME recognition

#### Preprocessing of video data

During the study, researchers captured two 15-min video clips, adding up to a total of 30 min. The 30-min video clip had 52 trials. In each of the 52 trials, participants were shown a fixation cross, followed by an audio-visual video stimulus lasting between 2 s and 1.5 min. They were later asked labeling and rating questions. These video segments included two kinds of noise: temporal and spatial (to focus only on the signal that is the Face of the participants, we need to delete any other information in the video that is noise). In order to pay attention to the participants' facial expressions, we initially reduced temporal noise by dividing the 30-min video into 52 parts, deleting portions with the fixation cross, label question, and rating question. This made sure that only the stimulus (video) parts were kept for additional examination. Every video segment was carefully edited by hand to preserve the accuracy and exactness of the stimulus display. Spatial noise, such as the rear monitor and other background elements, could be observed in every frame. The background Noise was removed by cropping the video to zoom in on the participant's face area. For this task, a custom Python program was written and used, which was carefully designed to focus on the area surrounding the face to ensure that it remained zoomed in and maintained a resolution of 480^*^640 pixels. Spatial and temporal noise were removed to prepare the videos for subsequent analysis stages, guaranteeing that only important data was handled.

## Feature selection and extraction

Feature selection is usually based on the Facial Action Coding System (FACS) framework. The FACS examines facial expressions by breaking them down into individual muscle movements called Action Units (AUs; Ekman and Friesen, 1978; Pfister et al., 2011). Here, we are using only relevant AUs which comes in the T-zone of the face area (that is the eye, eyebrow, nose, lips, and chin).

FACS coding is done either manually by trained experts who analyze facial movements frame by frame in a video or photograph, or by Automated Coding, which uses advances in technology that have led to the development of software that can automatically detect and code AUs from video input, increasing efficiency and consistency (Cohn and De la Torre, 2015). This study uses a **hybrid method** (both Manual and Automated) to select, extract, and classify ME data.

First, we used OpenFace 2.0 software toolkit (Baltrušaitis et al., 2016) to quantify AU's. Then, a handcrafted coding method based on quantified AU output was used for feature selection and ME snippet extraction. These snippets were cut based on time and emotion (Figure 1). Finally, two human coders frame-by-frame analyze the snippets and validate the presence of MEs.

**Figure 2:**
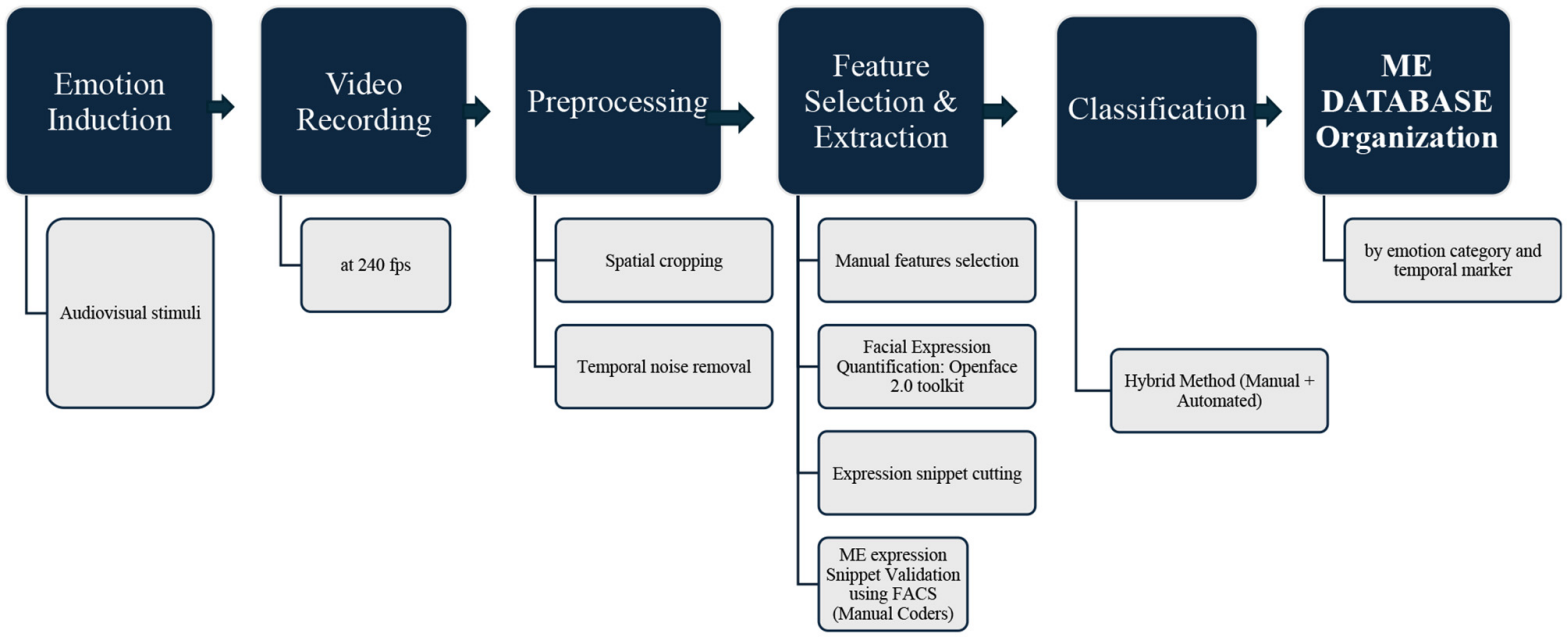
Workflow of ISMED dataset preparation: the pipeline of the Indian Spontaneous Micro-Expression Database (ISMED). The process includes (1) emotion induction via audiovisual stimuli, (2) high-speed video recording at 240 fps, (3) preprocessing with temporal noise removal and spatial cropping, (4) feature selection and extraction using manual feature coding and automated AU quantification (OpenFace 2.0), (5) validation of micro-expression snippets by trained coders using FACS, (6) classification with hybrid manual and automated methods, and (7) organization of validated micro-expression snippets by emotion category and temporal markers.

Although self-reports were collected from the 120 participants during the experiment, these were deliberately not used for labeling because participants were instructed to suppress their emotions and mask them with a neutral expression. Incorporating these self-reports would risk biasing the final labels. Thus, the definitive emotion annotations in ISMED are grounded in AU configurations and FACS-based manual validation, supported by balanced stimulus induction. The overall dataset construction pipeline is illustrated in Figure 2.

## Results

The Indian Spontaneous ME Database (ISMED) successfully addressed a critical gap in existing datasets by capturing a diverse range of spontaneous micro-expressions (MEs) from Indian participants, contrasting with the predominant focus on Western and East Asian populations in prior studies. Data from 120 participants (after excluding 7 due to technical issues) were analyzed using a hybrid approach that combined manual and automated techniques. High-resolution recordings ensured precise capture of subtle facial movements, while preprocessing steps, including temporal and spatial noise reduction via custom Python scripts, effectively isolated MEs for analysis.

A detailed evaluation revealed a consistent distribution of six basic emotions—happiness, sadness, anger, surprise, fear, and disgust—across participants. Manual validation demonstrated an accuracy rate exceeding 90%, underscoring the reliability of the dataset. When compared to other databases such as CASME and USF-HD, ISMED showcased unique cultural nuances inherent to Indian demographics, emphasizing the necessity for culturally sensitive datasets to enhance emotion recognition algorithms.

Table 2 presents the distribution of MEs across different time intervals and six basic emotions. Analysis revealed notable trends: positive emotions, particularly “Happy,” emerged as the most dominant and consistent across all time periods, with pronounced peaks in shorter durations (150–200 ms and 200–250 ms). This finding underscores the prevalence and rapid detectability of happiness in micro-expression studies. Similarly, “Angry” showed a steady increase in frequency as time intervals shortened, highlighting its rapid and intense manifestation in brief micro-expression windows. Conversely, “Disgust,” while moderately frequent, was primarily observed in mid-to-short durations, indicating it as a short-lived emotional response.

**Table 2:** Frequency distribution of six basic emotions (Angry, Disgust, Fear, Happy, Sad, Surprise) across time intervals in the ISMED dataset.

**Time period (ms)**	**Emotion**					
	**Angry**	**Disgust**	**Fear**	**Happy**	**Sad**	**Surprise**
450–500	1	3	–	5	1	1
400–450	3	6	–	28	2	–
350–400	4	3	–	48	–	4
300–3350	15	3	–	59	–	4
250–300	28	5	–	100	2	3
200–250	54	12	–	132	2	4
150–200	144	18	–	210	3	12

Note the dominance of short-duration expressions (150–250 ms), especially for happy and angry emotions.

While seven categories (including Neutral) were used during the rating task, the final ISMED dataset focuses on six basic emotions (Happy, Sad, Angry, Surprise, Fear, and Disgust). The Neutral category served as a baseline condition and was therefore not included in this table.

**Figure 3:**
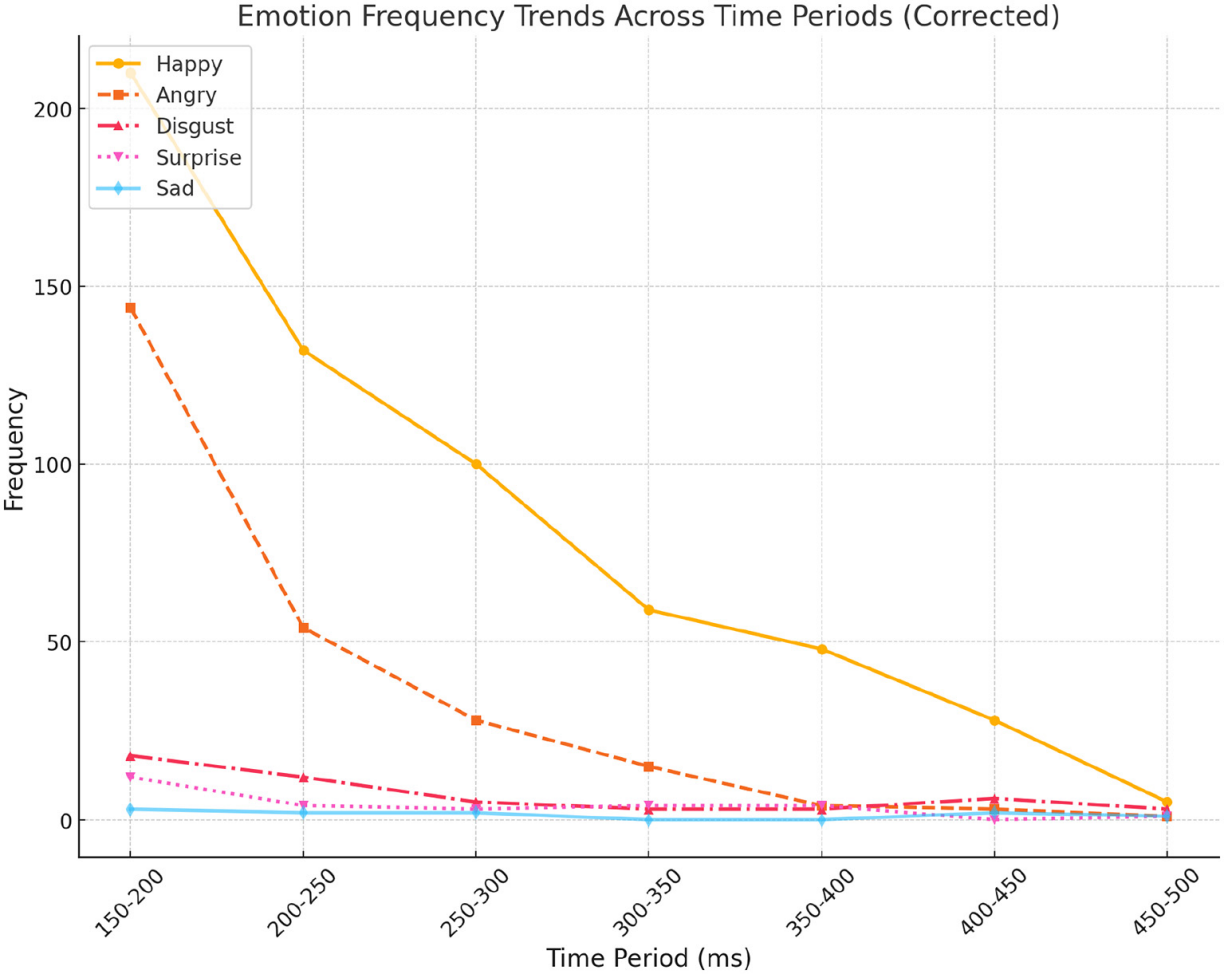
Area chart showing emotion frequency distribution across time intervals. “Happy” and “Angry” emotions are most dominant in shorter durations (150–250 ms), highlighting their rapid and frequent emergence in spontaneous micro-expressions.

In contrast, “Surprise” exhibited a sporadic presence, with slight peaks at 150–200 ms and 350–400 ms, suggesting its context-specific nature or limited representation in the dataset. “Sad” was notably sparse across all time intervals, indicating its subtlety in micro-expressions or potential underrepresentation in the sample. Interestingly, “Fear” was absent, raising questions about its detectability or contextual relevance within this study (Figure 3).

These findings further emphasize that emotional intensity is most prominent in shorter time periods, particularly under 200 ms, reinforcing the importance of high temporal resolution in micro-expression analysis. Additionally, the dominance of positive emotions like “Happy” over negative ones such as “Sad” or “Surprise” suggests potential contextual specificity or bias within the dataset. These insights highlight the importance of considering temporal dynamics and cultural nuances in micro-expression research, paving the way for refining detection methodologies for subtle or underrepresented emotions.

## Discussion

The aim of this study was to introduce the Indian Spontaneous ME Database (ISMED) as a culturally specific tool for understanding and analyzing MEs among Indian individuals. Our findings demonstrate that ISMED successfully captures a wide range of spontaneous MEs, revealing the cultural nuances inherent in the Indian demographic. By documenting these expressions, ISMED addresses a crucial gap in the current landscape of emotion databases, which predominantly feature Western or East Asian populations. This cultural specificity is vital for enhancing emotion recognition algorithms and making them adaptable and effective across diverse populations.

ISMED stands out by providing unique cultural insights that complement existing databases like CASME, USF-HD, Polikovsky, SAMM, etc. While these resources are invaluable for training models on posed or spontaneous expressions from other demographics, they fall short of encompassing the diversity needed to create truly global emotion recognition systems. Including Indian MEs in ISMED aligns with previous research (Matsumoto, 1993; Jack et al., 2009), emphasizing the need for culturally sensitive approaches to understanding emotional expression. By expanding the emotional repertoire and highlighting cultural influences on expression, ISMED contributes to refining existing theories of emotion, supporting the argument that emotion expression is not universally identical but deeply embedded in social and cultural contexts.

Methodologically, ISMED also offers advancements through its hybrid approach, combining manual and automated techniques to identify and validate MEs. This approach ensures that human expertise complements algorithmic precision, enhancing the reliability of ME detection. Such an integrated method provides a valuable model for future database development, illustrating how human intuition and computational efficiency can be merged effectively to capture complex emotional cues.

Practically, ISMED has significant applications in affective computing, clinical settings, and cross-cultural communication. By integrating ISMED into AI-driven systems, developers can create algorithms sensitive to Indian emotional cues, enhancing user interaction in AI-based services. In clinical contexts, ISMED offers psychologists a resource to better understand and diagnose emotional patterns specific to Indian individuals, potentially leading to culturally relevant therapeutic practices. Additionally, ISMED's insights into Indian MEs can aid in bridging communication gaps in multicultural interactions, providing a basis for developing globalized AI systems capable of interpreting diverse emotional expressions accurately.

However, the study is not without its limitations. The primary limitation is that the database does not capture fear expressions according to the Facial Action Coding System (FACS) framework. It is possible that the audio-visual stimuli used for emotion induction were not effective enough, or that a more thorough investigation is needed to identify any culturally specific Action Units (AUs) that may not align with the FACS framework. Additionally, while the controlled laboratory setting allows for standardization, it may not fully replicate the dynamics of real-world emotional expression. Although the ISMED dataset focuses on Indian individuals, it still lacks the diversity needed to capture India's regional and socio-economic variations. Furthermore, manual annotation is effective but also time-intensive, which may limit scalability, indicating the need for further refinement of automated coding methods.

To address these limitations, future research should expand ISMED to include a broader range of participants from different Indian regions and cultural backgrounds. Additionally, capturing MEs in more naturalistic settings could enhance the ecological validity of the database. Further development of automated algorithms that incorporate cultural insights from ISMED will be crucial in creating scalable models that adapt to cultural variations effectively. Interdisciplinary collaboration between psychologists, computer scientists, and cultural experts will be essential to achieve these objectives, ensuring that future emotion recognition systems are scientifically rigorous and culturally sensitive.

In conclusion, ISMED represents a significant step forward in the field of ME research, providing a culturally relevant tool that bridges theoretical and applied domains. Its impact extends beyond academic exploration, offering practical solutions for improving emotion recognition systems and contributing to the global understanding of human emotions in diverse cultural contexts.

## Data Availability

The datasets generated for this study are not publicly available due to their large size (~2 TB) and ethical constraints. However, the Indian Spontaneous Micro-Expression Database (ISMED), including raw and preprocessed video data, can be accessed under a controlled academic license upon formal written request to the corresponding author. The dataset is organized into folders by emotional category and temporal markers, with file names following the structure: Participant ID, Emotion Code, Stimulus Number, Frame Range (e.g., P000_H07_47_8995_46801.mp4). Custom code used for preprocessing and annotation (e.g., face cropping and AU extraction) will be made available by the corresponding author upon reasonable request, without undue reservation.

## References

[B1] Baltrušaitis T. Robinson P. Morency L. P. (2016). “Openface: an open source facial behavior analysis toolkit,” in 2016 IEEE Winter Conference on Applications of Computer Vision (WACV) (Lake Placid, NY: IEEE), 1–10. 10.1109/WACV.2016.7477553

[B2] Ben X. Ren Y. Zhang J. Wang S. J. Kpalma K. Meng W. . (2021). Video-based facial micro-expression analysis: a survey of datasets, features and algorithms. IEEE Trans. Pattern Anal. Machine Intell. 44, 5826–5846. 10.1109/TPAMI.2021.3067464 33739920

[B3] Cohn J. F. De la Torre F. (2015). “10 Automated face analysis for affective computing,” in The Oxford Handbook of Affective Computing, eds. R. A. Calvo, S. K. D'Mello, J. Gratch, and A. Kappas (New York, NY: Oxford University Press) 131–150. 10.1093/oxfordhb/9780199942237.013.013

[B4] Davison A. K. Lansley C. Costen N. Tan K. Yap M. H. (2016). Samm: a spontaneous micro-facial movement dataset. IEEE Trans. Affect. Comput. 9, 116–129. 10.1109/TAFFC.2016.2573832

[B5] Ekman P. (2003). Emotions Revealed: Recognizing Faces and Feelings to Improve Communication and Emotional Life. New York, NY: Times Books.

[B6] Ekman P. (2006). Darwin and Facial Expression: A Century of Research in Review (Expanded 3rd ed.). Cambridge, MA: Malor Books.

[B7] Ekman P. Friesen W. V. (1969). Nonverbal leakage and clues to deception. Psychiatry 32, 88–106. 10.1080/00332747.1969.11023575 5779090

[B8] Ekman P. Friesen W. V. (1978). Facial Action Coding System (FACS) [Database record]. APA PsycTests. 10.1037/t27734-000

[B9] Jack R. E. Garrod O. G. B. Schyns P. G. (2009). Dynamic facial expressions of emotion transmit an evolving hierarchy of signals over time. Curr. Biol. 19, 1687–1693. 10.1016/j.cub.2013.11.064 24388852

[B10] Li X. Pfister T. Huang X. Zhao G. Pietikäinen M. (2013). “A spontaneous micro-expression database: Inducement, collection and baseline,” in 2013 10th IEEE International Conference and Workshops on Automatic Face and Gesture Recognition (fg) (IEEE), 1–6.

[B11] Matsumoto D. (1993). Ethnic differences in affect intensity, emotion judgments, display rule attitudes, and self-reported emotional expression in an American sample. Motiv. Emot. 17, 107–123. 10.1007/BF00995188

[B12] Matsumoto D. (2006). Are cultural differences in emotion regulation mediated by personality traits? J. Cross Cult. Psychol. 37, 421–437. 10.1177/0022022106288478

[B13] Matsumoto D. Hwang H. C. (2011). Reading facial expressions of emotion. Psychol. Sci. Agenda 25. 10.1037/e574212011-002. [Epub ahead of print].

[B14] Matsumoto D. Hwang H. S. Yamada H. (2012). Cultural differences in the relative contributions of face and context to judgments of emotions. J. Cross Cult. Psychol. 43, 198–218. 10.1177/0022022110387426

[B15] Pfister T. Li X. Zhao G. Pietikäinen M. (2011). “Recognising spontaneous facial micro-expressions,” in Proceedings of the IEEE International Conference on Computer Vision (ICCV) (Barcelona: IEEE), 1449–1456. 10.1109/ICCV.2011.6126401

[B16] Polikovsky S. Kameda Y. Ohta Y. (2009). “Facial micro-expressions recognition using high-speed camera and 3D-gradient descriptor,” in Proceedings of the 3rd International Conference on Imaging for Crime Detection and Prevention (ICDP 2009) (London: IET), 1–6. 10.1049/ic.2009.0244

[B17] Qu F. Wang S. J. Yan W. J. Li H. Wu S. Fu X. (2017). CAS (ME)^2^: a database for spontaneous macro-expression and micro-expression spotting and recognition. IEEE Trans. Affect. Comput. 9, 424–436. 10.1109/TAFFC.2017.2654440

[B18] Radlak K. Bozek M. Smolka B. (2015). “Silesian deception database: Presentation and analysis.” in Proceedings of the 2015 ACM Workshop on Multimodal Deception Detection (WMDD '15) (Seattle, WA: Association for Computing Machinery (ACM SIGCHI)), 29–35. 10.1145/2823465.2823469

[B19] Shreve M. Godavarthy S. Goldgof D. Sarkar S. (2011). “Macro-and micro-expression spotting in long videos using spatio-temporal strain,” in 2011 IEEE International Conference on Automatic Face and Gesture Recognition (FG) (Santa Barbara, CA: IEEE), 51–56. 10.1109/FG.2011.5771451

[B20] Vinciarelli A. Dielmann A. Favre S. Salamin H. (2009). “Canal9: a database of political debates for analysis of social interactions,” in 2009 3rd International Conference on Affective Computing and Intelligent Interaction and Workshops (Amsterdam: IEEE), 1–4. 10.1109/ACII.2009.5349466

[B21] Warren G. Schertler E. Bull P. (2009). Detecting deception from emotional and unemotional cues. J. Nonverbal Behav. 33, 59–69. 10.1007/s10919-008-0057-733234008

[B22] Yan W. Wu Q. Liu Y. Wang S. Fu X. (2014b). CASME II: an improved spontaneous micro-expression database and the baseline evaluation. PLoS ONE 9:e86041. 10.1371/journal.pone.0086041 24475068 PMC3903513

[B23] Yan W. J. Wu Q. Liu Y. J. Wang S. J. Fu X. (2014a). CASME database: a dataset of spontaneous micro-expressions collected from neutralized faces. IEEE Trans. Affect. Comput. 4, 149–163. 10.1109/FG.2013.6553799

